# Identification and Analysis of the Plasma Membrane H^+^-ATPase Gene Family in Cotton and Its Roles in Response to Salt Stress

**DOI:** 10.3390/plants13243510

**Published:** 2024-12-16

**Authors:** Cong Cheng, Fengyuan Zhang, Li Li, Zhiyong Ni

**Affiliations:** 1 Xinjiang Key Laboratory for Ecological Adaptation and Evolution of Extreme Environment Biology, College of Life Sciences, Xinjiang Agricultural University, Urumqi 830052, China; cheng_c@xjau.edu.cn (C.C.); lili@xjau.edu.cn (L.L.); 2 College of Life Science, Xinjiang Agricultural University, Urumqi 830052, China; 13872027155@163.com

**Keywords:** PM H^+^-ATPase gene family, cotton species, bioinformatics, salt stress, qRT-PCR

## Abstract

Plant plasma membrane (PM) H^+^-ATPase functions as a proton-motive force by exporting cellular protons to establish a transmembrane chemical gradient of H^+^ ions and an accompanying electrical gradient. These gradients are crucial for plant growth and development and for plant responses to abiotic and biotic stresses. In this study, a comprehensive identification of the PM H^+^-ATPase gene family was conducted across four cotton species. Specifically, 14 genes were identified in the diploid species *Gossypium arboreum* and *Gossypium raimondii*, whereas 39 and 43 genes were identified in the tetraploid species *Gossypium hirsutum* and *Gossypium barbadense*, respectively. The characteristics of this gene family were subsequently compared and analyzed using bioinformatics. Chromosomal localization and collinearity analyses elucidated the distribution characteristics of this gene family within the cotton genomes. Gene structure and phylogenetic analyses demonstrated the conservation of this family across cotton species, whereas the examination of *cis*-acting elements in gene promoters highlighted their involvement in environmental stress and hormone response categories. An expression profile analysis revealed eight genes whose expression was upregulated under salt stress conditions, and quantitative real-time PCR results suggested that the cotton PM H^+^-ATPase genes may play crucial roles in conferring resistance to salt stress. These findings establish a robust foundation for subsequent investigations into the functions of cotton PM H^+^-ATPase genes and may offer valuable insights for selecting genes for resistance breeding programs.

## 1. Introduction

Plasma membrane (PM) H^+^-ATPase generates a proton-motive force by actively transporting protons out of the cell, thereby establishing both a transmembrane chemical gradient of H^+^ ions and an accompanying electrical gradient [1]. Consequently, the PM H^+^-ATPase plays a pivotal role in establishing membrane potential and pH homeostasis while also generating the proton-motive force necessary for the transmembrane transport of various substances. Thus, it is a key enzyme in plant growth, development, and stress resistance [2]. The PM H^+^-ATPase is a member of the P3A family of proton pumps within the P-type ATPases superfamily, which is exclusively found in plants and fungi [3]. On the basis of phylogenetic analysis, PM H^+^-ATPase can be categorized into five distinct subfamilies (I–V). Since the first plant PM H^+^-ATPase was characterized, extensive screening of this gene family has been conducted across various plant species, including *Arabidopsis thaliana* (*AHA1*-*AHA12*) [4] and *Oryza sativa* (*OSA1*-*OSA10*) [5]. Research on plant PM H^+^-ATPase has been progressively advancing across various fields, highlighting its distinctive characteristics and biological functions.

PM H^+^-ATPase is a monomeric protein composed of a single polypeptide chain of fewer than 1000 amino acid residues, resulting in a functional unit with an approximate molecular weight of 100 kDa [3]. It is capable of oligomerizing to form dimeric and hexameric complexes [6] but still has a minimalist structure compared with the proton pump of the large multi-subunit F_O_F_1_ mitochondrial ATPase [7]. The PM H^+^-ATPase comprises ten transmembrane segments (TM1–TM10) along with the N- and C-terminal [3]. Structurally, it is organized into five distinct domains: the A- (actuator), N- (nucleotide-binding), P- (phosphorylation), R- (regulation), and N-terminal domains [8]. Among these domains, the P domain, which encompasses the catalytic phosphorylated aspartate within the canonical sequence DKTGTLT, is crucial for catalytic activity and is therefore commonly employed as the characteristic sequence for gene identification [9]. The C-terminal region, which includes the R domain, has been demonstrated to function as an autoinhibitory domain [10]. The expression of plant PM H^+^-ATPase genes predominantly exhibits specific spatiotemporal patterns, with distinct vital functions [4]. Through the cloning of genes in various plant species and examination of their evolutionary relationships, it has been demonstrated that plant PM H^+^-ATPase is related to its counterparts in fungi and P-type ATPases in animals, indicating its evolutionary conservation [11].

The PM H^+^-ATPase is crucial for plant growth, development, and stress resistance. The proton motive force generated by the PM H^+^-ATPase is essential for the uptake of nutrients in plants, including ammonium (NH_4_^+^) [12] and nitrate (NO_3_^−^) [13] as nitrogen (N) sources, as well as H_2_PO_4_^−^ and HPO_4_^2−^ for phosphorus (P) absorption [14]. The activation of PM H^+^-ATPase can be stimulated by both blue and red light, leading to increased stomatal opening and consequently modulating plant gas exchange and photosynthesis processes [15]. By enhancing photosynthetic efficiency and nutrient uptake, PM H^+^-ATPase has the potential for applications aimed at improving crop yield [16]. PM H^+^-ATPase is responsive to a variety of abiotic stresses, such as salinity [17], drought [18], and temperature fluctuations [19]. Notably, in salt tolerance, the proton gradient established by the PM H^+^-ATPase is indispensable for the functioning of the Salt Overly Sensitive 1 (SOS1) sodium transporter [20]. Several proteins, including SOS2-like protein5 (PKS5) and SOS3-like calcium-binding protein 1 (SCaBP1), function as negative regulators or inhibitors of PM H^+^-ATPase under normal conditions. In response to salt stress, this inhibitory effect is alleviated, resulting in the activation of PM H^+^-ATPase and an increase in plant salt tolerance [21]. Nonetheless, further research is needed to elucidate the mechanisms by which plants perceive salt stress and subsequently regulate PM H^+^-ATPase activity.

Cotton (*Gossypium* spp.) is a vital cash crop that is valued for its elongated seed fibers and substantial edible oil content. The *Gossypium* genus consists of 45 diploid species (2n = 2× = 26) and 7 tetraploid species (2n = 4× = 52) [22]. Among these, four independently domesticated cotton species, *G. hirsutum* (tetraploid), *G. barbadense* (tetraploid), *G. arboreum* (diploid), and *G. raimondii* (diploid), have undergone in-depth research in recent decades. The first cotton genome of *G. raimondii* was sequenced and assembled in 2012 because it has the smallest nuclear genome. To date, over 44 cotton genomes have been documented, 33 of which have been sequenced in the past five years [23]. The continuous improvement of genome sequencing has laid a foundation for the breeding of cotton varieties and the investigation of gene functions [24]. In 2018, the cotton P-type ATPase superfamily was identified and analyzed across four distinct species, revealing that several P-type ATPase genes potentially contribute to cotton growth and fiber development, as indicated by their expression profiles [25]. In mepiquat chloride-induced K^+^ uptake in cotton, the PM H^+^-ATPase mediates plasma membrane hyperpolarization and activates the K^+^ channels of cotton roots [26]. Under the conditions of low available phosphorus in the soil, the activity of the cotton PM H^+^-ATPase enzyme is reduced, thus limiting fiber elongation [27].

The findings presented above highlight the primary roles of this gene family in cotton. Current research predominantly addresses the roles of PM H^+^-ATPase in nutrient absorption and fiber elongation in cotton, with a notable absence of studies examining its involvement in stress resistance. Furthermore, advancements in cotton genome sequencing suggest that the gene family identification reported in 2018 may now be insufficient to satisfy contemporary research demands. Consequently, there is a need for a comprehensive re-evaluation of the cotton PM H^+^-ATPase gene family to facilitate a more precise investigation of its function in stress resistance. In this study, the PM H^+^-ATPase gene family was identified in four cotton species, and a comprehensive analysis of their chromosomal distribution, gene structure, evolutionary relationships, and expression profiles was conducted. Key genes that respond to salt stress were screened via an expression profile analysis and validated using quantitative real-time PCR (qRT-PCR). The findings from this study provide a foundation for future investigations into the functional roles of the PM H^+^-ATPase genes in cotton.

## 2. Results

### 2.1. Identification of the Cotton PM H^+^-ATPase Gene Family

To identify the cotton PM H^+^-ATPase genes, a total of 197 plant PM H^+^-ATPase gene nucleotide sequences were retrieved from the NCBI database. These sequences were subsequently aligned against the genomes of four different cotton species, resulting in the identification of 43, 39, 14, and 14 putative genes from *G. barbadense*, *G. hirsutum*, *G. arboreum*, and *G. raimondii*, respectively. A domain analysis using the SMART tool confirmed that most of these genes possessed the P domain and the PS00154 motif. Several genes lacking the P domain and PS00154 were also identified as members of this family of genes because of their high sequence similarity and homology with other known genes, as well as being annotated as PM H^+^-ATPase in the genome database. All of the PM H^+^-ATPase genes were renamed according to their chromosomal distribution order, following the naming conventions in *Arabidopsis* as *AHA*. Detailed information and sequences of the cotton *AHA* genes are provided in Table S1. The average protein length was 735 amino acids in *G. barbadense*, 759 amino acids in *G. hirsutum*, and 946 amino acids in both *G. arboreum* and *G. raimondii*. Their protein length varied from 111 amino acids (*GbAHA39* and *GhAHA36*) to 987 amino acids (*GrAHA01*). The predicted molecular weight (MW) and isoelectric point (pI) of each PM H^+^-ATPase gene are also detailed in Table S1. The prediction of subcellular localizations indicates that all the cotton PM H^+^-ATPase genes are located on the plasma membrane, which is consistent with the functional localization of plant PM H^+^-ATPase genes.

### 2.2. Chromosomal Distribution and Gene Collinearity

**Figure 1:**
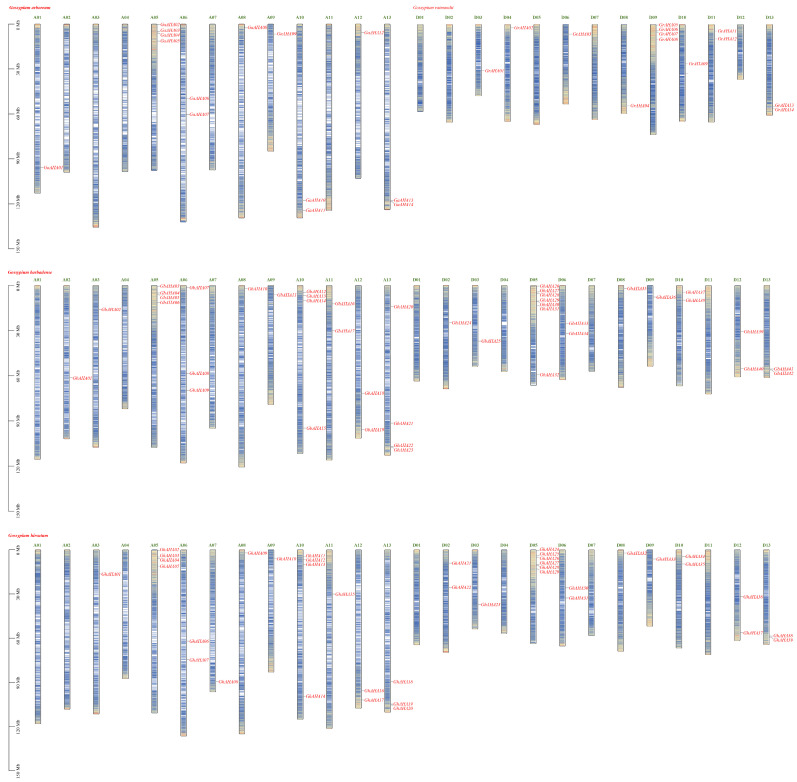
Chromosome distribution of the cotton PM H^+^-ATPase gene family. The cotton *AHA* genes were distributed in the genomes of *G. barbadense*, *G. hirsutum*, *G. arboreum*, and *G. raimondii*. The *AHA* gene name is highlighted with a red font, and the heatmap within the genome bar shows the gene density of the chromosome. The scale on the left of the genome represents the length of the chromosome.

The distribution of cotton AHA genes according to chromosomal distribution data from multiple genomic databases is depicted in Figure 1. In *G. hirsutum*, 39 *AHA* genes are distributed across 19 chromosomes, among which D05 harbors 6 *AHA* genes, and chromosomes A05 and A10 each contain 4 genes. The remaining 16 chromosomes possess only one or two *AHA* genes, whereas chromosomes A01, A02, A04, D01, D04, D07, and D11 do not contain any. Similarly, in *G. barbadense*, 42 *AHA* genes are also distributed across 19 chromosomes, with chromosomes D05, A05, and A10 containing the greatest number of genes. The gene *GbAHA43* is located not on chromosomes but on a scaffold. In *G. arboreum*, 14 genes were mapped across eight chromosomes, with chromosome A05 containing 4 *AHA* genes. In *G. raimondii*, 14 genes were distributed across eight chromosomes, with chromosome D09 harboring 4 *AHA* genes.

**Figure 2:**
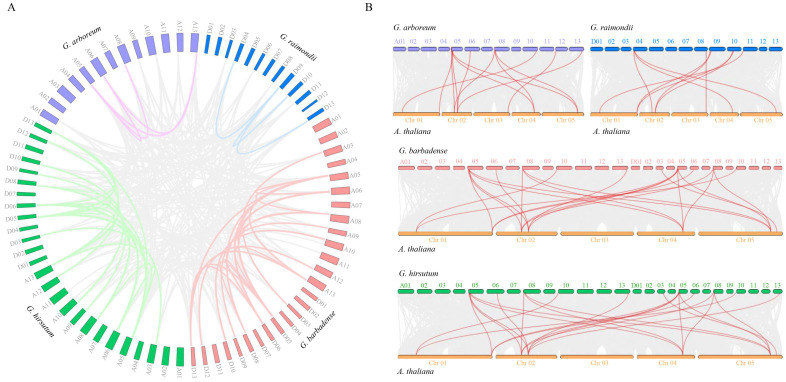
Collinearity analysis of cotton *AHA* genes across multiple genomes. (**A**) Collinearity analysis of the *AHA* duplicated gene pairs in *G. hirsutum*, *G. barbadense*, *G. arboretum*, and *G. raimondii*. The syntenic *AHA* gene pairs within the cotton genomes are shown with different colored lines, and those between the cotton genomes are shown as grey lines. (**B**) Collinearity analysis of the *AHA* gene pairs of cotton with *Arabidopsis*.

A gene collinearity analysis revealed the presence of 358 duplicated *AHA* gene pairs among the genomes of the four cotton species. The number of lineal/parallel homologous duplicated *AHA* gene pairs was 88 for *Gh*-*Gb*, 48 for *Gh*-*Ga*, 42 for *Gh*-*Gr*, 41 for *Gb*-*Gr*, 47 for *Gb*-*Ga*, and 21 for *Ga*-*Gr*. Additionally, 31 of the collinear gene pairs within the genomes were in *Gh*-*Gh*, 31 in *Gb*-*Gb*, 6 in *Ga*-*Ga*, and 3 in *Gr*-*Gr* (Figure 2A). In light of the evolutionary relationships among species, an analysis of the collinearity between *Arabidopsis* and various cotton genomes was conducted. The findings revealed the presence of 23, 22, 12, and 9 *AHA* gene pairs exhibiting a high degree of collinearity in the *Gh*-*At*, *Gb*-*At*, *Ga*-*At*, and *Gr*-*At* comparisons, respectively (Figure 2B). These results suggest that segmental duplication and whole-genome duplication may serve as the primary driving forces behind the amplification of the cotton *AHA* gene family.

### 2.3. Motif Prediction and Gene Structure

To elucidate the gene structure of cotton *AHA* genes, a comprehensive analysis was conducted using 110 AHA proteins identified from four cotton cultivars. The conserved motifs were predicted via the MEME online tool, with the findings presented in Figure S1. Ten conserved motifs, ranging in length from 29 to 50 amino acids, were identified. Notably, motifs 1, 3, 4, and 6 were present in more than 100 *AHA* genes, whereas the remaining motifs were observed in at least 83 AHA proteins. These results validate the accuracy of the identification of cotton *AHA* genes and underscore the conservation of cotton AHA proteins.

**Figure 3:**
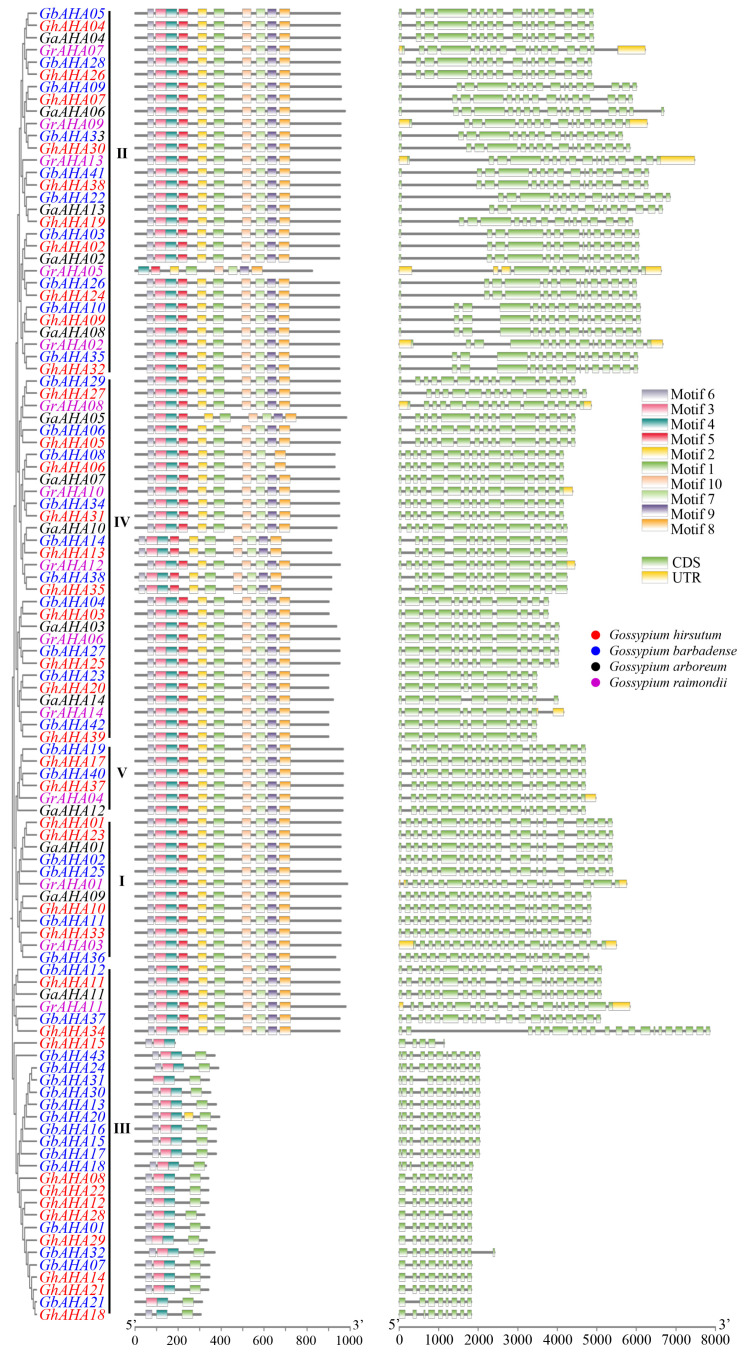
The structure of the cotton *AHA* genes. Clustering was conducted via MEGA, and the genes identified from different cultivars are shown in different colors. The roman numerals represent the type that genes belong to. The conserved motifs on the left were obtained via MEME, which corresponds to Figure S1, and the bar below refers to the amino acid length of the AHA protein. The exon–intron patterns on the right are summarized from the genome annotation file, and the bar below shows the length of the nucleic acid fragment where the gene is located.

On the basis of the motif prediction results presented above, the exon–intron patterns of the genes derived from genome annotation were visualized in conjunction with the phylogenetic tree to elucidate the structural characteristics of cotton AHA proteins (Figure 3). The *AHA* genes identified from various cotton species are distinguished by different color codes for their names. All the cotton *AHA* genes can be categorized into five types on the basis of their structural and phylogenetic characteristics, with the number of genes in each type being 12, 30, 29, 30, and 6 for types I through V, respectively. The cotton *AHA* genes present an average of 17 exons per gene, and only some type III genes have fewer than 10 exons because of their short gene length. Most type II AHA genes contain a long intron following the first exon. Moreover, the 3′ UTR of the 13 *GrAHA* genes was annotated, and the 5′ UTR of the 9 *GrAHA* genes was annotated. These findings suggest the complex structure of the cotton *AHA* genes, especially the large number of introns, which may facilitate increased alternative splicing events and may also explain why type III has 22 short-sequence *AHA* genes.

### 2.4. Phylogenetic Relationship and Synonymous and Non-Synonymous Substitution Rates

**Figure 4:**
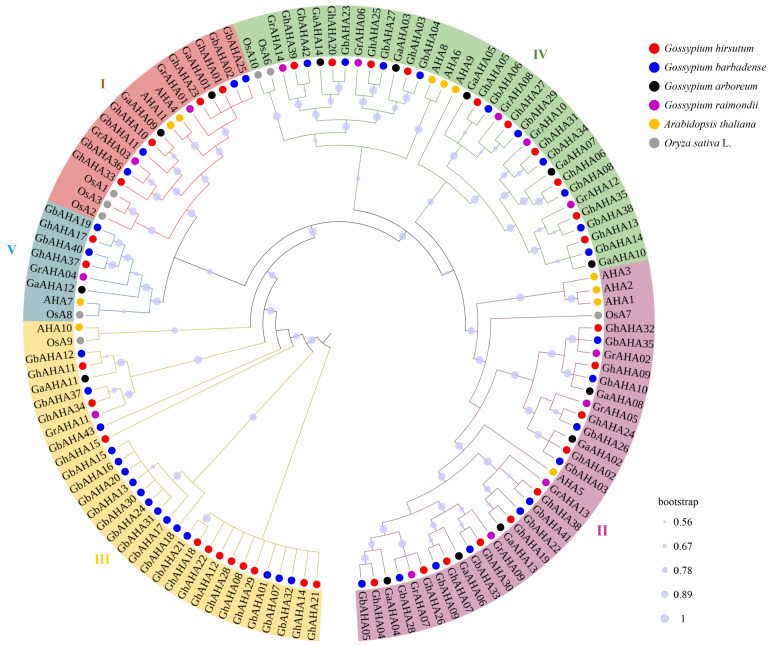
The phylogenetic tree of the cotton *AHA* gene family. The color under the genes and the roman numerals represent the type to which they belong, and the round dots before the gene name represent the plant species. The phylogenetic tree was constructed via MEGA with the neighbor–joining method. The substitution model was the Tamura 3-parameter model, and the bootstrap value was 2000.

The phylogenetic tree of the cotton *AHA* family was constructed from 107 protein sequences via the neighbor-joining (NJ) method, excluding *GbAHA39*, *GbAHA16*, and *GhAHA36* because of their insufficient length. Additionally, the *AHA* genes from *Arabidopsis* and rice were incorporated to facilitate the classification of the family types (refer to Table S2). The resulting phylogenetic tree is depicted in Figure 4, where the high bootstrap values indicate the tree’s accuracy. On the basis of prior studies in *Arabidopsis*, members of the *AHA* family were categorized into five distinct types, I–V. The number of cotton *AHA* genes from different species that were distributed on each type was 12, 30, 29, 30, and 6 from type I to type V, respectively. Notably, these genes from various cotton species predominantly clustered within the same clade, which was distinct from the clustering of the *Arabidopsis* and rice genes. This finding suggests considerable genetic divergence between the cotton *AHA* genes and those of *Arabidopsis* and rice, potentially indicating an independent evolutionary trajectory for the cotton *AHA* gene family. This divergence may offer insights into the novel functional roles of these genes.

The Ka/Ks ratio was computed to evaluate the selection pressure on *AHA* collinear gene pairs in different cotton cultivars. A total of 71 gene pairs identified through collinearity analysis were utilized for this investigation, with results obtained for 62 of these pairs (refer to Table S3). The Ka/Ks ratio for all of the examined gene pairs was less than one, suggesting that all of the *AHA* genes in cotton have been subjected to purifying selection throughout their evolutionary history.

### 2.5. cis-Acting Elements in the Cotton AHA Gene Promoter Region

**Figure 5:**
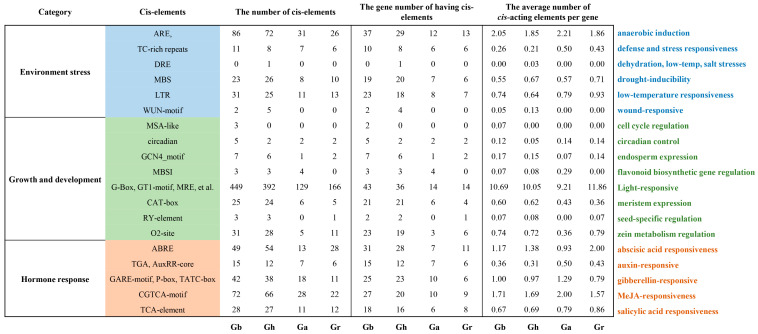
The *cis*-acting element analysis of the cotton *AHA* gene promoter.

The *cis*-acting elements within the gene promoter sequence play crucial roles in influencing gene expression and regulation. To gain a deeper understanding of the expression characteristics of cotton *AHA* genes, a 2 kb region upstream of the *AHA* transcription start site in the genomic DNA was analyzed to identify the *cis*-acting elements. The findings were categorized and are presented statistically in Figure 5. The numbers of *cis*-acting elements identified in the cotton *AHA* gene promoters of *G. barbadense*, *G. hirsutum*, *G. arboreum*, and *G. raimondii* were 885, 729, 281, and 321, respectively. In the categories of environmental stress, the *cis*-acting elements of cotton *AHA* genes are associated with anaerobic induction, defense and stress responsiveness, drought inducibility, and low-temperature responsiveness. Within the growth and development categories, eight distinct types of *cis*-acting elements were identified, with the light-responsive type being the most prevalent. Conversely, in the five categories related to the hormone response, the *cis*-acting elements are distributed relatively uniformly across numerous promoters of the cotton *AHA* genes. These findings indicate that the expression of the *AHA* genes is predominantly regulated by environmental stress and hormone responses rather than the growth and development pathways.

To facilitate a more comprehensive comparison among the various cotton cultivars, the average number of *cis*-acting elements per gene was employed as a metric for assessing differences. Notably, the *AHA* genes in *G. raimondii* exhibit the highest average number of 22.93 *cis*-acting elements per gene upstream, with a significant proportion associated with drought inducibility, low-temperature responsiveness, light responsiveness, zein metabolism regulation, abscisic acid responsiveness, and salicylic acid responsiveness compared with the other three cotton cultivars. Moreover, the average number of *cis*-acting elements located upstream of the *G. arboretum AHA* genes was the lowest, with an average of 20.07 elements per gene, but their ratios of anaerobic induction, defense and stress responsiveness, flavonoid biosynthesis-related gene regulation, auxin-responsiveness, gibberellin-responsiveness, and MeJA-responsiveness were greater than those of the other three cotton cultivars. These findings suggest that the expression of the *G. arboretum AHA* genes may be more responsive to environmental and hormonal influences. Furthermore, compared with those in the other two cultivars, the AHA genes in *G. barbadense* and *G. hirsutum* presented a greater number of *cis*-acting elements associated with wound response and meristem expression. This observation suggests that the superior disease resistance and growth characteristics of these species may be partially attributable to the functional role of *AHA* genes.

### 2.6. Expression Pattern Analysis

**Figure 6:**
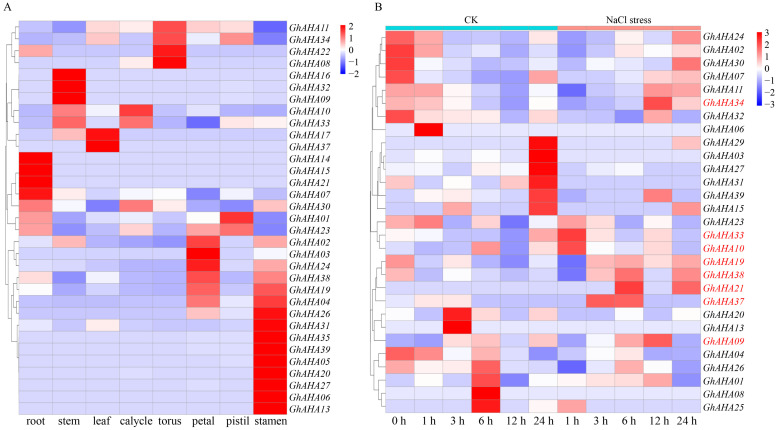
The expression patterns of cotton *AHA* genes in multiple tissues (**A**) and under NaCl stress (**B**). The genes that respond to stress are highlighted in red in the heatmap.

The transcriptome of *G. hirsutum* (TM-1) was used as the database for analyzing the expression patterns of the cotton *AHA* genes. The expression profiles of the *GhAHA* genes across various tissues and under salt stress conditions were visualized through heatmaps. A total of 33 *GhAHA* genes exhibited pronounced tissue-specific expression patterns, whereas the remaining 6 genes were not expressed. Notably, nearly all of the genes demonstrated high expression levels in a single tissue (Figure 6A). Among the *GhAHA* genes, approximately half were predominantly expressed in reproductive organs, including the stamen, pistil, and petal. These findings underscore the indispensable role of the *AHA* gene family in the growth and development of cotton. Under NaCl stress, the expression patterns of the cotton *AHA* genes were more complicated (Figure 6B). During a 24-h NaCl treatment, 29 *GhAHA* genes were expressed, with the majority showing high expression levels at various time points under control conditions. Notably, only eight genes were upregulated in response to the NaCl treatment. Among these genes, *GhAHA33* and *GhAHA10* presented the most rapid response, with upregulation observed as early as one hour after the NaCl treatment. Following 3 h of NaCl treatment, the expression levels of *GhAHA19*, *GhAHA37*, and *GhAHA38* were upregulated and remained elevated for a period of time. The expression of *GhAHA21* increased under both the 6-h and 24-h treatments, whereas the expression of *GhAHA34* and *GhAHA09* increased under the 12-h NaCl treatment. These eight genes are likely to play crucial roles in the response of cotton plants to NaCl stress, and their functions warrant further investigation through in-depth research.

### 2.7. The Role of AHA Genes in Response to NaCl Stress in Cotton

**Figure 7:**
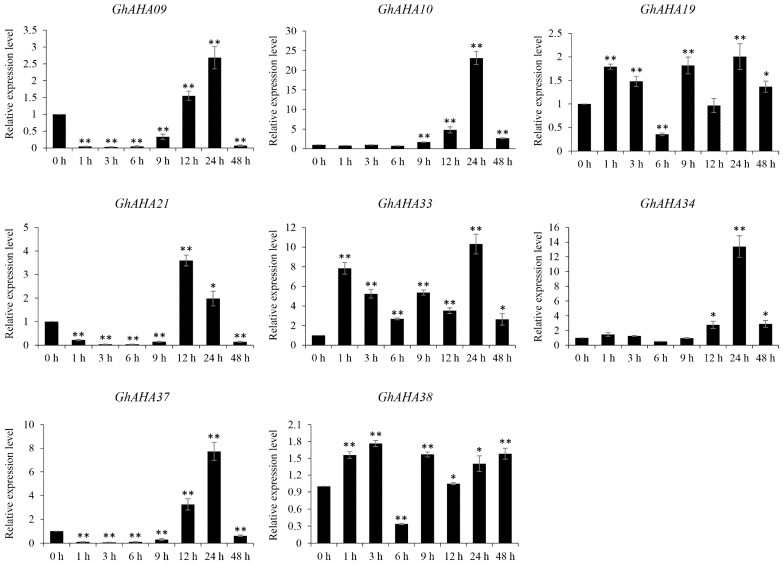
Relative expression levels of *GhAHA* genes in cotton leaves subjected to different durations of NaCl stress. “*” indicates two-tailed significance of *p* ≤ 0.05, “**” indicates two-tailed significance of *p* ≤ 0.01.

To validate the role of cotton *AHA* genes in response to NaCl-induced stress, qRT-PCR was used to assess the relative expression levels of the eight genes selected above. The cotton seedlings were treated with 150 mM NaCl to simulate a salt stress environment, in which the morphophysiological characteristics of the cotton plants included accelerated cotyledon shedding, turning yellow, decreased plant height and fresh weight, and growth inhibition but not death. After various durations of stress, samples were collected from multiple tissues, including leaves and roots, for a gene expression analysis. The results of the relative expression levels of these genes in the cotton leaves are presented in Figure 7. These eight genes were all significantly upregulated after salt stress, which is consistent with the expression analysis results of the transcriptome expression profiles. However, two distinct expression patterns were observed among these eight genes. Specifically, the relative expression levels of *GhAHA09*, *GhAHA21*, and *GhAHA37* significantly decreased at the onset of salt treatment (before 9 h), followed by significant upregulation between 12 and 24 h compared with those in the control (CK), after which they subsequently significantly decreased again at 48 h. The genes *GhAHA10* and *GhAHA34* presented similar expression patterns but remained unchanged at the beginning of the salt treatment and subsequently became upregulated between 12 and 48 h. In contrast, although the gene expression levels of *GhAHA19, GhAHA33*, and *GhAHA38* continuously fluctuated and even significantly decreased after 6 h of treatment, they were significantly upregulated from the beginning of the salt treatment, and their high expression was maintained for almost 48 h. Among these eight genes, *GhAHA10* presented the greatest relative change in expression level, exceeding a 20-fold increase, followed by *GhAHA34*, *GhAHA33*, and *GhAHA37*. However, in cotton roots, the expression of these genes was generally downregulated following salt stress, except for that of *GhAHA09*, whose expression was upregulated more than 20-fold after 12 h of treatment (Figure S2). These findings demonstrate a positive response of the *AHA* genes to salt stress in the cotton leaves, in contrast with a negative response in the roots. These findings validate the functional role of these genes in response to salt stress at the expression level and suggest that their function is dependent on the biological processes occurring in cotton leaves. These insights will provide guidance and a reference for future in-depth investigations into the functional verification of these cotton *AHA* genes.

## 3. Discussion

PM H^+^-ATPase is necessary for plant growth, development, and stress defense. In this study, the PM H^+^-ATPase gene family was screened from four cotton species and comprehensively analyzed. The investigation identified 110 cotton PM H^+^-ATPase genes including 14 in diploid cotton and approximately 40 in tetraploid cotton. However, a 2018 study on the cotton P-ATPase gene family reported the identification of 48 PM H^+^-ATPase genes across all four cultivars, comprising 14 genes in *G. raimondii*, 22 in *G. hirsutum*, 12 in *G. barbadense*, and none in *G. arboreum* [25]. This study identified 110 genes in the same four cotton species, with 62 additional genes missed by the previous study. These findings clearly underscore the need for more extensive identification efforts. In contrast to the research progress in 2018, 33 novel cotton genome databases have been reported, which established a foundation for more comprehensive gene identification and characteristic analysis. Owing to the particularity of cotton species, which have different diploid and tetraploid genomes, the comparative analysis of gene families among species has become meaningful. This approach can provide reference information for the process of chromosome reduplication and further anchor gene function through differences in characteristics between species; for example, the comprehensive identification and expression analysis of the *B-Box* gene family was recently completed among the four cotton species [28].

The 14 PM H^+^-ATPase genes identified in diploid cotton in this study are similar to the number found in other plant species, such as the 12 genes of *Arabidopsis* [4], 10 of *Oryza sativa* [5], 13 of *Helianthus annuus* [9], and 9 of *Salvia miltiorrhiza* [29]. However, the more than 40 genes identified in tetraploid cultivar cotton is much more than the number of genes found in other tetraploid species, such as 28 genes in *Triticum aestivum* (wheat) [30] and 32 genes in *Brassica napus* [31]. The number of genes in tetraploid cotton is more than twice that in diploid cotton, indicating that the cotton PM H^+^-ATPase gene family may have undergone expansion during genome duplication. Some of the PM H^+^-ATPase genes in tetraploid cotton may perform functions that do not involve encoding enzymes. For example, 15 PM H^+^-ATPase genes in *G. barbadense* and 11 genes in *G. hirsutum* are much shorter than the others. The average length of these 26 genes was only 320 amino acids, whereas the average length of the other genes was 945 amino acids. This phenomenon did not occur in diploid cotton. Through sequence homology alignment, collinearity analysis, and P-domain searches, these short genes were confirmed to belong to this family, but they are often expressed only in certain specific situations, such as some kinds of abiotic stress. These results indicate that some special regulatory functions may have evolved in the *AHA* gene family of tetraploid cotton, which is worthy of further research.

Coding genes typically perform their functions through translation into proteins, but their position and clustering on chromosomes also reflect their functional patterns to some extent [32]. The gene chromosomal distribution results obtained in this study indicate that the cotton PM H^+^-ATPase genes are commonly located at the ends of chromosomes with high gene density. In particular, at the end of chromosome 05, there are 4–6 *AHA* genes in both Group A and Group D chromosomes. These clustered genes are not homologous, nor do they have collinearity characteristics, so they are not continuously replicated copied genes. This phenomenon may suggest a specific regulatory mechanism in clustered *AHA* genes. In addition, the chromosomal localization characteristics of the PM H^+^-ATPase genes in this study are completely inconsistent with those reported in 2018, which showed that the *AHA* gene was clustered at chromosome segment number 09. Owing to the recent improvements in cotton genome data and the comprehensive identification in this study, the chromosomal distribution characteristics of cotton *AHA* obtained here are more reliable and can lay the foundation for in-depth functional research.

The gene structure analysis revealed the conservation of the cotton PM H^+^-ATPase gene family in terms of both the motif prediction in MEME and their intron–exon structures. The cotton *AHA* gene has an average of 17 exons per gene, which is larger than that in other plants, such as an average of 13 exons in sunflowers [9]. An evolutionary analysis also revealed that the *AHA* gene in cotton is conserved and has a distant relationship with those in *Arabidopsis* and rice. This result is consistent with research on sunflowers [9]. Previous reports have shown that the *AHA* gene evolved before the separation of monocotyledons and dicotyledons, indicating strong independent evolution among different species [4]. The Ka/Ks results indicate that the family has undergone strong purifying selection during evolution. Therefore, combining the analysis results from both the structural and evolutionary perspectives, the cotton *AHA* family is relatively conserved and independent.

The analysis of *cis*-acting elements on gene promoters revealed that the PM H^+^-ATPase genes in cotton are highly regulated by many *cis*-acting factors involved in the light response, environmental stress, and hormone response. Among these, light-responsive *cis*-acting elements constitute the largest proportion, approximately 10%, of the cotton *AHA* gene promoter. Previous reports have shown that PM H^+^-ATPase activation is a type of photosynthesis-dependent process [33,34]. Therefore, the abundance of light-responsive *cis*-acting elements may reflect the high activity of cotton PM H^+^-ATPase. Furthermore, a comparison of the proportions of *cis*-acting elements among different cotton species revealed that *AHA* genes are regulated differently among species; for example, *G. arboreum* is more susceptible to environmental and hormonal influences.

PM H^+^-ATPase, as an energy-consuming center responsible for establishing electrochemical gradients and proton dynamics, is directly related to the absorption of nutrients and stress resistance in plants [1]. Through extensive hormone regulation, plants rapidly adjust their growth and stress resistance status. This is especially true for the acid growth theory hypothesis for auxin-mediated cell expansion. PM H^+^-ATPase activation results in plasma membrane hyperpolarization, thus promoting solute and water uptake and driving cell expansion [35]. In *Arabidopsis*, the PM H^+^-ATPase is regulated by auxin and modulates primary root elongation by mediating H^+^ efflux in the root elongation zone [36]. However, the analysis of the cotton *AHA* gene promoters revealed numerous *cis*-acting elements involved in the abscisic acid response rather than auxin-response elements, which is consistent with research on sunflowers [9] and *Brassica napus* [31]. These results may provide new directions for research on the response of *AHA* genes to hormones.

The expression analysis revealed that most PM H^+^-ATPase genes in cotton have specific spatiotemporal expression patterns, as they are expressed in specific tissues or are induced under specific stresses. Interestingly, the tissue-specific expression of PM H^+^-ATPase in the P-type ATPase gene family has been previously reported [37]. In particular, the specific upregulated gene expression of some *AHA* genes in cotton sexual organs is consistent with research in *Nicotiana plumbaginifolia*, which reported that increased PM H^+^-ATPase gene expression may be related to the rapid and asymmetric development of flower organ cells [38].

Under salt stress, the expression of eight PM H^+^-ATPase genes increased after different durations of stress according to the genome database. For further validation, qRT-PCR was conducted on these genes, and the results revealed that the expression of these eight genes was upregulated after salt stress in cotton leaves, but there were significant differences in their expression patterns. The genes encoding *GhAHA19*, *GhAHA33*, and *GhAHA38* were upregulated immediately after exposure to stress, and the genes encoding *GhAHA10* and *GhAHA34* were upregulated after 9 h of stress, indicating that the response of cotton to salt follows a gradient. In contrast, the changes in the expression of *GhAHA09*, *GhAHA21*, and *GhAHA37* were more noteworthy. These genes were significantly downregulated in the early stage of salt stress and then significantly upregulated again, which is a rather special phenomenon. The expression of these three genes ultimately does not reach a particularly high level, being significantly lower than the highest expression of *AHA10* and *AHA34*. We believe that the proton pumps formed by these *AHA* proteins may have different utilization efficiencies and specific regulatory mechanisms. The proteins encoded by the *GhAHA09*, *GhAHA21*, and *GhAHA37* genes are not essential proton pumps that perform core functions.

At the beginning of salt stress, the inhibition of ATP synthesis by salt stress leads to a decrease in cellular energy, resulting in the suppression of the expression of these three genes to reduce energy consumption. However, over time, the intracellular salt concentration increases. To accelerate the efflux of Na ions and slow down the toxic effects, cells begin to specifically regulate and significantly increase their expression levels. This differential regulation of different genes under the same conditions forms the salt tolerance regulatory mechanism of cotton. Physiologically, through this process, cell energy is allocated more reasonably, and the harmful effects of salt stress on plant cells are effectively alleviated, which has important physiological significance. Moreover, this phenomenon significantly occurs in leaf tissues, as photosynthesis in leaves is more severely affected by salt stress, and ATP synthesis is more strongly inhibited. However, in root tissue, hydroponic culture conditions lead to reduced oxygen availability and decreased ATP generation. Consequently, the efflux of sodium ions may be limited by the PM H^+^-ATPase on the plasma membrane, resulting in the inhibition of the expression of most AHA genes. Among the eight genes studied, *GhAHA09* was upregulated in both cotton leaves and roots under salt stress, and *GhAHA10* expression increased more than 20-fold in cotton leaves. Therefore, further research and verification will be conducted on the above two genes in the future. In sunflowers, *HHA4* and *HHA11*, which were screened via expression analysis under salt stress, were confirmed to improve plant salt resistance via transgenic *Arabidopsis* plants [9]. The findings obtained in this study lay the foundation for functional research on the cotton PM H^+^-ATPase gene family and provide a valuable reference for exploring the involvement of this family in plant stress resistance.

## 4. Materials and Methods

### 4.1. Identification of the P-Type Plasma Membrane H^+^-ATPase Family in Cotton

The genome sequences of *G. barbadense* (Hai7124_HAU, version 1.1), *G. hirsutum* (TM-1_ZJU, version 2.1), *G. arboretum* (A2_CRI, version 1.0), and *G. raimondii* (JGI, version 2.1) were obtained from COTTONOMICS (http://cotton.zju.edu.cn/index.htm) (accessed on 3 August 2024) [39], Cotton MD (https://yanglab.hzau.edu.cn/CottonMD.1) (accessed on 3 August 2024) [40], and COTTONGEN (https://www.cottongen.org/) (accessed on 3 August 2024) [41]. The nucleotide sequences of the plasma membrane H^+^-ATPase genes of different species were downloaded from the NCBI Nucleotide database (https://www.ncbi.nlm.nih.gov) (accessed on 9 August 2024) and used as queries to blast against the cotton genome (*E* value < 1.0 × 10^−6^). The candidate genes obtained were then used to identify the P domain (DKTGT[L/I/V/M][T/I]) and ProSite PS00154 with the web tools Pfam (http://pfam-legacy.xfam.org/) and SMART. After verification, the coding sequences and protein sequences of the cotton PM H^+^-ATPase genes were downloaded from the genome database and renamed by the naming conventions in *Arabidopsis thaliana* as *AHA*, such as *GbAHA*. The 2 kb sequences upstream of the cotton *AHA* genes were simultaneously downloaded. The molecular weights (MWs) and isoelectric points (pIs) of the gene proteins were predicted via the ExPASy website (https://www.expasy.org/) (accessed on 20 August 2024), the physicochemical parameters were calculated with the web tool ProtParam (https://web.expasy.org/protparam/) (accessed on 20 August 2024), and the subcellular localizations were predicted via the Plant-mPLoc database (http://www.csbio.sjtu.edu.cn/bioinf/plant-multi/) (accessed on 20 August 2024).

### 4.2. Chromosomal Distribution and Gene Duplication

The chromosomal distribution information of the *AHA* genes was obtained from the cotton genome and was visualized via the TBtools software (version 2.118) [42]. The genome of *Arabidopsis* was downloaded from the TAIR database (https://www.arabidopsis.org/) (accessed on 15 August 2024). The collinear blocks of genes among the different cotton genomes and those within the Arabidopsis genome were analyzed via one-step MCScanX and visualized with the Dual Synteny Plot of TBtools software.

### 4.3. Gene Structure and Motif Prediction

The exon–intron patterns of the *AHA* genes were analyzed on the basis of the *GFF* gene annotations of the genomes. The conserved motif was predicted via MEME online tools with default parameters (http://meme-suite.org/tools/meme) (accessed on 11 August 2024). The gene structure was visualized via TBtools, which uses the phylogenetic trees constructed via MEGA (version 7.0.26) as the gene grouping criteria.

### 4.4. Phylogenetic Analysis and Estimation of the Ka/Ks Ratios of the Duplicated Pairs

The multiple sequence alignment and phylogenetic tree construction of the cotton AHA proteins were conducted via MEGA (version 7.0.26) with the neighbor–joining (NJ) method. The bootstraps (2000) were set to evaluate the reliability of the branches. The *AHA* genes of *Arabidopsis* and rice obtained from the TAIR database and the Rice Gene Index (RGI) database (https://riceome.hzau.edu.cn/) (accessed on 12 August 2024) [43] were added to the phylogenetic tree to cluster the different types of *AHA* genes. The Evolview online tool (http://www.evolgenius.info/evolview/) (accessed on 19 August 2024) was used to enhance the appearance of the phylogenetic tree.

The duplicated *AHA* gene pairs from different cotton genomes were used to calculate the synonymous (Ka) and non-synonymous (Ks) substitution values, and the Ka/Ks ratio was estimated via the Ka/Ks Calculator online tool (http://services.cbu.uib.no/tools/kaks/) (accessed on 17 August 2024).

### 4.5. cis-Acting Element Analysis of the PM H^+^-ATPase Gene Promoter Region

The 2000 bp sequences of the cotton PM H^+^-ATPase gene promoters were extracted from multiple cotton genome databases and used for *cis*-acting element detection via the Plant CARE database (https://bioinformatics.psb.ugent.be/webtools/plantcare/html/) (accessed on 18 August 2024) [44]. The *cis*-acting elements obtained were tabulated and statistically analyzed to determine the number of different types, the *AHA* gene number of *cis*-acting elements, and the average number of *cis*-acting elements per *AHA* gene.

### 4.6. Expression Pattern Analysis

The expression profiles of the *G. hirsutum AHA* genes, including their expression in different tissues and under multiple durations of salt stress, were downloaded from the database of the cotton genome. The expression patterns of these *GhAHA* genes were visualized via the TBtools software.

### 4.7. Role of Cotton AHA Genes in Response to Salt Stress

The seeds of *G. hirsutum* were soaked in water for two hours and then germinated in a moist environment for two days. The germinated seeds were transferred to moist vermiculite for cultivation and then to hydroponics with Hoagland solution after the first leaf grew. The culture conditions included a temperature of 27 ± 1 °C and a 16/8 photoperiod (light/dark) following the protocol used in a previous report [45]. After 2 weeks of growth, the seedlings were stressed with 150 mM NaCl dissolved in the Hoagland solution [46,47]. After treatment times of 0 h, 1 h, 3 h, 6 h, 9 h, 12 h, 24 h, and 48 h, the first leaves and roots of the cotton plants were collected and stored at −80 °C. Total RNA was extracted from the cotton plant samples with an RNA Pure Plant Kit (Takara, Dalian, China) and reverse transcribed by using a PrimeScript RT Reagent Kit (Takara, Dalian, China). The gene-specific primers used were designed with Primer Premier 5 (Table S4). qRT-PCR was conducted on a LightCycler 96 Instrument (Roche Inc., Branchburg, NJ, USA) with TB Green Premix Ex Taq^TM^ II (Takara, Dalian, China). The *GhActin* gene was used as the reference gene [48], and the relative expression levels of the *GhAHA* genes were calculated via the 2^−ΔΔCT^ method.

## 5. Conclusions

In summary, the PM H^+^-ATPase gene family includes 14, 14, 39, and 43 members in the cotton species *G. arboreum*, *G. raimondii*, *G. hirsutum*, and *G. barbadense*, respectively. This gene family has a high degree of conservation across these cotton species in terms of gene structure and phylogenetics and may be regulated by various biological processes, especially responses to environmental stress and hormones. A transcriptome analysis revealed the tissue-specific expression patterns of these genes, among which eight genes were verified by qRT–PCR to be upregulated under salt stress. These genes may play a key role in the salt tolerance mechanism of cotton. These results provide an important basis for understanding the H^+^-ATPase gene family and cotton stress resistance breeding.

## Data Availability

All the data generated during this study are included within the article or its Supplementary Materials.

## Supplementary Materials

The following supporting information can be downloaded at https://www.mdpi.com/article/10.3390/plants13243510/s1. Figure S1: The conserved motif prediction of the cotton PM H^+^-ATPase gene family by MEME. The prediction was conducted by default parameters. The height of each letter represents the conservation of amino acids. Figure S2: The relative expression level of *GhAHA* genes in cotton root treated with different durations of salt stress. “*” indicates two-tailed significance of *p* ≤ 0.05, “**” indicates two-tailed significance of *p* ≤ 0.01. Table S1: The information on the cotton PM H^+^-ATPase gene family. Table S2: The genes from out-group species in the phylogenetic tree of the cotton PM H^+^-ATPase family. Table S3: The Ka/Ks values of gene pairs in cotton AHA. Table S4: The primers used for qRT-PCR (5′-3′) to verify the function of the cotton PM H^+^-ATPase genes in response to salt stress.
