# Supplementary material for: Identification and Analysis of the Plasma Membrane H+-ATPase Gene Family in Cotton and Its Roles in Response to Salt Stress
Source: Plants (Basel). 2024 Dec 16;13(24):3510. doi: 10.3390/plants13243510 (PMC11728463; doi:10.3390/plants13243510)
Supplement: Supplementary file 1 [file plants-13-03510-s001.zip › Table. S1-revised.pdf]

Table S1. The information on the cotton PM H^{+}-ATPase gene family.

Genome	Gene name	Accession NO.	Group	Protein length	Molecular weight MW (Da)	pI	Subcellular location
G. barbadense (Hai-7124)	GbAHA01	GB_A02G1271	III	346	38533.73	8.13	Plasma membrane
	GbAHA02	GB_A03G0771	I	956	105317.24	6.23	Plasma membrane
	GbAHA03	GB_A05G0042	II	951	104714.69	6.66	Plasma membrane
	GbAHA04	GB_A05G0521	IV	901	98590.37	5.44	Plasma membrane
	GbAHA05	GB_A05G0661	II	954	104969.7	6.38	Plasma membrane
	GbAHA06	GB_A05G1228	IV	953	105088.78	5.83	Plasma membrane
	GbAHA07	GB_A06G0143	III	346	38343.44	6.96	Plasma membrane
	GbAHA08	GB_A06G1259	IV	929	102486.84	6.64	Plasma membrane
	GbAHA09	GB_A06G1354	II	958	105870.52	6.41	Plasma membrane
	GbAHA10	GB_A08G0234	II	951	104819.72	6.48	Plasma membrane
	GbAHA11	GB_A09G0270	I	956	105358.4	6.34	Plasma membrane
	GbAHA12	GB_A10G0501	III	952	105042.42	5.51	Plasma membrane
	GbAHA13	GB_A10G0618	III	378	41874.31	6.34	Plasma membrane
	GbAHA14	GB_A10G0713	IV	913	100234.08	5.87	Plasma membrane
	GbAHA15	GB_A10G2014	III	378	42036.49	7.01	Plasma membrane
	GbAHA16	GB_A11G1192	III	378	41847.29	6.34	Plasma membrane
	GbAHA17	GB_A11G2022	III	378	41808.23	6.96	Plasma membrane
	GbAHA18	GB_A12G1216	III	332	36747.28	5.65	Plasma membrane
	GbAHA19	GB_A12G2443	V	968	106468.48	8.37	Plasma membrane
	GbAHA20	GB_A13G0707	III	393	43500.25	6.13	Plasma membrane
	GbAHA21	GB_A13G1592	III	313	34780.23	6.96	Plasma membrane

	GbAHA22	GB_A13G2216	II	954	105165.89	7.22	Plasma membrane
	GbAHA23	GB_A13G2275	IV	899	97911.36	5.06	Plasma membrane
	GbAHA24	GB_D02G1124	III	388	43030.64	6.47	Plasma membrane
	GbAHA25	GB_D03G1061	I	956	105259.21	6.3	Plasma membrane
	GbAHA26	GB_D05G0043	II	951	104673.59	6.66	Plasma membrane
	GbAHA27	GB_D05G0516	IV	952	104304.76	5.51	Plasma membrane
	GbAHA28	GB_D05G0655	II	954	104957.67	6.38	Plasma membrane
	GbAHA29	GB_D05G1216	IV	953	104917.62	5.89	Plasma membrane
	GbAHA30	GB_D05G1255	III	351	38818.68	5.93	Plasma membrane
	GbAHA31	GB_D05G1561	III	345	38196.79	6.12	Plasma membrane
	GbAHA32	GB_D05G3722	III	371	41156.65	7.58	Plasma membrane
	GbAHA33	GB_D06G1233	II	957	105766.48	6.41	Plasma membrane
	GbAHA34	GB_D06G1361	IV	951	104833.64	6.99	Plasma membrane
	GbAHA35	GB_D08G0240	II	951	104861.76	6.48	Plasma membrane
	GbAHA36	GB_D09G0238	I	932	102702.3	6.31	Plasma membrane
	GbAHA37	GB_D10G0514	III	952	104854.17	5.44	Plasma membrane
	GbAHA38	GB_D10G0873	IV	913	100373.33	6.08	Plasma membrane
	GbAHA39	GB_D12G1060	/	111	12671.71	4.31	Plasma membrane
	GbAHA40	GB_D12G2448	V	968	106436.35	7.31	Plasma membrane
	GbAHA41	GB_D13G2165	II	954	105155.85	7.22	Plasma membrane
	GbAHA42	GB_D13G2223	IV	899	97962.48	5.08	Plasma membrane
	GbAHA43	GB_scaffold678_objG0002	III	371	40843.08	5.23	Plasma membrane
G. hirsutum (TM-1)	GhAHA01	GH_A03G0779	I	956	105331.27	6.23	Plasma membrane
	GhAHA02	GH_A05G0035	II	951	104714.69	6.66	Plasma membrane
	GhAHA03	GH_A05G0518	IV	901	98638.55	5.6	Plasma membrane
	GhAHA04	GH_A05G0658	II	954	104969.7	6.38	Plasma membrane

GhAHA05	GH_A05G1216	IV	953	105102.8	5.83	Plasma membrane
GhAHA06	GH_A06G1217	IV	929	102702.1	6.98	Plasma membrane
GhAHA07	GH_A06G1310	II	958	105882.58	6.41	Plasma membrane
GhAHA08	GH_A07G2188	III	342	37838.79	6.02	Plasma membrane
GhAHA09	GH_A08G0234	II	951	104789.7	6.48	Plasma membrane
GhAHA10	GH_A09G0241	I	956	105372.43	6.34	Plasma membrane
GhAHA11	GH_A10G0500	III	952	104981.36	5.51	Plasma membrane
GhAHA12	GH_A10G0613	III	342	37900.02	6.54	Plasma membrane
GhAHA13	GH_A10G0696	IV	913	100217.09	5.87	Plasma membrane
GhAHA14	GH_A10G1891	III	346	38480.71	8.12	Plasma membrane
GhAHA15	GH_A11G1997	III	187	20528.77	4.83	Plasma membrane
GhAHA16	GH_A12G1918	/	154	16697.23	5.48	Plasma membrane
GhAHA17	GH_A12G2352	V	968	106526.52	8.37	Plasma membrane
GhAHA18	GH_A13G1479	III	306	34449.05	8.54	Plasma membrane
GhAHA19	GH_A13G2082	II	954	105155.85	7.22	Plasma membrane
GhAHA20	GH_A13G2140	IV	899	97925.38	5.06	Plasma membrane
GhAHA21	GH_D02G0671	III	342	38087.21	7.62	Plasma membrane
GhAHA22	GH_D02G1076	III	342	37846	7.62	Plasma membrane
GhAHA23	GH_D03G1041	I	956	105331.27	6.23	Plasma membrane
GhAHA24	GH_D05G0039	II	951	104673.59	6.66	Plasma membrane
GhAHA25	GH_D05G0516	IV	952	104267.7	5.5	Plasma membrane
GhAHA26	GH_D05G0656	II	954	104971.7	6.38	Plasma membrane
GhAHA27	GH_D05G1217	IV	953	104917.62	5.89	Plasma membrane
GhAHA28	GH_D05G1251	III	323	36015.95	6.96	Plasma membrane
GhAHA29	GH_D05G1552	III	335	37217.21	6.96	Plasma membrane
GhAHA30	GH_D06G1184	II	957	105798.46	6.41	Plasma membrane

	GhAHA31	GH_D06G1313	IV	951	104806.61	6.99	Plasma membrane
	GhAHA32	GH_D08G0248	II	951	104861.76	6.48	Plasma membrane
	GhAHA33	GH_D09G0238	I	956	105307.31	6.24	Plasma membrane
	GhAHA34	GH_D10G0526	III	952	104853.23	5.53	Plasma membrane
	GhAHA35	GH_D10G0888	IV	913	100408.37	6.12	Plasma membrane
	GhAHA36	GH_D12G1023	/	111	12671.71	4.31	Plasma membrane
	GhAHA37	GH_D12G2367	V	968	106436.35	7.31	Plasma membrane
	GhAHA38	GH_D13G2062	II	954	105155.85	7.22	Plasma membrane
	GhAHA39	GH_D13G2125	IV	899	98000.58	5.11	Plasma membrane
G. arboreum (A2-CRI)	GaAHA01	Ga01G1999	I	856	105331.27	6.23	Plasma membrane
	GaAHA02	Ga05G0036	II	951	104714.69	6.66	Plasma membrane
	GaAHA03	Ga05G0540	IV	937	102906.31	5.59	Plasma membrane
	GaAHA04	Ga05G0688	II	954	104985.7	6.28	Plasma membrane
	GaAHA05	Ga05G1275	IV	983	108635.97	5.79	Plasma membrane
	GaAHA06	Ga06G1246	II	977	107894.91	6.32	Plasma membrane
	GaAHA07	Ga06G1340	IV	951	104929.76	6.99	Plasma membrane
	GaAHA08	Ga08G0278	II	951	104789.7	6.48	Plasma membrane
	GaAHA09	Ga09G0253	I	956	105372.43	6.34	Plasma membrane
	GaAHA10	Ga10G2237	IV	938	103273.66	6.16	Plasma membrane
	GaAHA11	Ga10G2558	III	952	104960.3	5.48	Plasma membrane
	GaAHA12	Ga12G0644	V	966	106065.93	7.05	Plasma membrane
	GaAHA13	Ga13G2250	II	954	105155.85	7.22	Plasma membrane
	GaAHA14	Ga13G2313	IV	921	100734.68	5.3	Plasma membrane
G. raimondii (D5-JGI)	GrAHA01	Gorai.003G094300	I	987	108901.51	6.21	Plasma membrane
	GrAHA02	Gorai.004G027300	II	951	104861.76	6.48	Plasma membrane
	GrAHA03	Gorai.006G023600	I	956	105311.32	6.24	Plasma membrane

GrAHA04	Gorai.008G232800	V	966	106080.94	6.86	Plasma membrane
GrAHA05	Gorai.009G004200	II	825	90865.71	8.67	Plasma membrane
GrAHA06	Gorai.009G053500	IV	952	104279.71	5.51	Plasma membrane
GrAHA07	Gorai.009G068100	II	956	105169.96	6.38	Plasma membrane
GrAHA08	Gorai.009G125200	IV	953	104989.69	5.83	Plasma membrane
GrAHA09	Gorai.010G123000	II	957	105793.48	6.38	Plasma membrane
GrAHA10	Gorai.010G136300	IV	949	104682.43	6.79	Plasma membrane
GrAHA11	Gorai.011G053900	III	980	108032.95	5.44	Plasma membrane
GrAHA12	Gorai.011G089100	IV	953	105065.57	6.09	Plasma membrane
GrAHA13	Gorai.013G212200	II	954	105213.89	6.95	Plasma membrane
GrAHA14	Gorai.013G217900	IV	899	97976.51	5.08	Plasma membrane
