# Supplementary material for: Identification and Analysis of the Plasma Membrane H+-ATPase Gene Family in Cotton and Its Roles in Response to Salt Stress
Source: Plants (Basel). 2024 Dec 16;13(24):3510. doi: 10.3390/plants13243510 (PMC11728463; doi:10.3390/plants13243510)
Supplement: Supplementary file 1 [file plants-13-03510-s001.zip › Table. S2.pdf]

Table S2. The genes from out-group species in the phylogenetic tree of cotton PM H^+-ATPase family.

Species type	Species	Gene name	Gene ID	Type
Monocots	*Oryza sativa*	OsA1	OsAzu_03g0030590	I
	Oryza sativa	OsA2	OsAzu_07g0005880	I
	Oryza sativa	OsA3	OsAzu_12g0022280	I
	Oryza sativa	OsA5	OsAzu_11g0014310	II
	Oryza sativa	OsA6	OsAzu_02g0037050	IV
	Oryza sativa	OsA7	OsAzu_04g0029400	II
	Oryza sativa	OsA8	OsAzu_03g0000120	V
	Oryza sativa	OsA9	OsAzu_03g0006230	III
	Oryza sativa	OsA10	OsAzu_06g0005760	IV
Eudicots	*Arabidopsis thaliana*	AHA1	AT2G18960	II
	Arabidopsis thaliana	AHA2	AT4G30190	II
	Arabidopsis thaliana	AHA3	AT5G57350	II
	Arabidopsis thaliana	AHA4	AT3G47950	I
	Arabidopsis thaliana	AHA5	AT2G24520	II
	Arabidopsis thaliana	AHA6	AT2G07560	IV
	Arabidopsis thaliana	AHA7	AT3G60330	V
	Arabidopsis thaliana	AHA8	AT3G42640	IV
	Arabidopsis thaliana	AHA9	AT1G80660	IV
	Arabidopsis thaliana	AHA10	AT1G17260	III
	Arabidopsis thaliana	AHA11	AT5G62670	I
