# Supplementary material for: Identification and Analysis of the Plasma Membrane H+-ATPase Gene Family in Cotton and Its Roles in Response to Salt Stress
Source: Plants (Basel). 2024 Dec 16;13(24):3510. doi: 10.3390/plants13243510 (PMC11728463; doi:10.3390/plants13243510)
Supplement: Supplementary file 1 [file plants-13-03510-s001.zip › Table. S3-revised.pdf]

Table S3. The Ka/Ks values of gene pairs in cotton AHA.

Gene pairs		Ka	Ks	Ka/Ks	*P*-Value (Fisher)
GhAHA01	*GhAHA23*	0.0000	0.0426	0.0010	0
GhAHA09	*GhAHA02*	0.0213	0.6594	0.0323	1.01×10^{-172}
GhAHA02	*GhAHA24*	0.0015	0.0421	0.0345	4.51×10^{-16}
GhAHA02	*GhAHA32*	0.0204	0.6525	0.0312	1.79×10^{-172}
GhAHA04	*GhAHA07*	0.0282	0.3255	0.0866	6.55×10^{-78}
GhAHA04	*GhAHA19*	0.0292	0.4406	0.0662	1.24×10^{-110}
GhAHA04	*GhAHA26*	0.0033	0.0329	0.1010	3.00×10^{-10}
GhAHA04	*GhAHA30*	0.0287	0.3182	0.0902	2.69×10^{-75}
GhAHA05	*GhAHA27*	0.0023	0.0420	0.0550	1.10×10^{-14}
GhAHA06	*GhAHA31*	0.0060	0.0415	0.1437	1.24×10^{-9}
GhAHA07	*GhAHA26*	0.0286	0.3368	0.0849	4.24×10^{-81}
GhAHA07	*GhAHA30*	0.0033	0.0249	0.1322	1.76×10^{-7}
GhAHA09	*GhAHA24*	0.0219	0.6897	0.0318	8.18×10^{-178}
GhAHA09	*GhAHA32*	0.0038	0.0327	0.1154	1.52×10^{-9}
GhAHA10	*GhAHA33*	0.0038	0.0472	0.0803	8.44×10^{-15}
GhAHA11	*GhAHA34*	0.0072	0.0574	0.1260	1.57×10^{-14}
GhAHA13	*GhAHA35*	0.0060	0.0312	0.1915	6.17×10^{-7}
GhAHA17	*GhAHA37*	0.0052	0.0336	0.1547	3.41×10^{-8}
GhAHA19	*GhAHA26*	0.0294	0.4518	0.0650	2.16×10^{-113}
GhAHA19	*GhAHA30*	0.0312	0.4455	0.0700	4.64×10^{-110}
GhAHA20	*GhAHA25*	0.1009	0.7028	0.1436	7.74×10^{-120}
GhAHA20	*GhAHA39*	0.0077	0.0473	0.1629	1.06×10^{-9}
GhAHA24	*GhAHA32*	0.0210	0.6833	0.0307	1.60×10^{-177}
GhAHA25	*GhAHA39*	0.1014	0.6719	0.1508	3.00×10^{-113}
GhAHA26	*GhAHA30*	0.0290	0.3245	0.0892	3.50×10^{-77}
GhAHA26	*GhAHA38*	0.0297	0.4392	0.0677	5.65×10^{-110}
GhAHA30	*GhAHA38*	0.0311	0.4128	0.0754	5.54×10^{-101}
GbAHA02	*GbAHA25*	0.0010	0.0419	0.0231	6.70×10^{-18}
GbAHA03	*GbAHA10*	0.0223	0.6599	0.0338	2.24×10^{-172}
GbAHA03	*GbAHA26*	0.0020	0.0382	0.0511	7.91×10^{-14}
GbAHA03	*GbAHA35*	0.0208	0.6593	0.0316	8.41×10^{-173}
GbAHA05	*GbAHA09*	0.0287	0.3299	0.0869	2.01×10^{-78}
GbAHA05	*GbAHA22*	0.0297	0.4420	0.0672	3.17×10^{-110}
GbAHA05	*GbAHA28*	0.0033	0.0310	0.1077	1.34×10^{-9}
GbAHA05	*GbAHA33*	0.0291	0.3181	0.0916	1.17×10^{-74}
GbAHA05	*GbAHA41*	0.0296	0.4285	0.0690	3.45×10^{-107}
GbAHA06	*GbAHA29*	0.0019	0.0383	0.0484	3.92×10^{-14}

GbAHA08	*GbAHA34*	0.0049	0.0433	0.1142	4.24×10^{-11}
GbAHA09	*GbAHA28*	0.0291	0.3371	0.0863	1.96×10^{-80}
GbAHA09	*GbAHA33*	0.0052	0.0233	0.2236	2.09×10^{-5}
GbAHA10	*GbAHA26*	0.0224	0.6684	0.0336	1.04×10^{-172}
GbAHA10	*GbAHA35*	0.0042	0.0320	0.1314	8.44×10^{-9}
GbAHA11	*GbAHA36*	0.0048	0.0463	0.1032	8.49×10^{-13}
GbAHA12	*GbAHA37*	0.0073	0.0566	0.1283	2.78×10^{-14}
GbAHA14	*GbAHA38*	0.0059	0.0295	0.1996	2.08×10^{-6}
GbAHA19	*GbAHA40*	0.0038	0.0333	0.1138	1.75×10^{-9}
GbAHA22	*GbAHA28*	0.0299	0.4580	0.0654	3.00×10^{-115}
GbAHA22	*GbAHA41*	0.0014	0.0372	0.0390	3.03×10^{-14}
GbAHA23	*GbAHA27*	0.0994	0.7096	0.1401	2.48×10^{-122}
GbAHA26	*GbAHA35*	0.0210	0.6676	0.0314	4.88×10^{-173}
GbAHA27	*GbAHA42*	0.0991	0.6689	0.1482	1.82×10^{-113}
GbAHA28	*GbAHA33*	0.0294	0.3222	0.0912	7.31×10^{-76}
GbAHA28	*GbAHA41*	0.0298	0.4368	0.0681	3.19×10^{-109}
GbAHA33	*GbAHA41*	0.0315	0.4148	0.0761	8.32×10^{-101}
GaAHA04	*GaAHA06*	0.0282	0.3336	0.0846	2.36×10^{-80}
GaAHA02	*GaAHA08*	0.0213	0.6585	0.0323	2.55×10^{-173}
GaAHA03	*GaAHA13*	0.1201	3.7586	0.0320	0
GaAHA04	*GaAHA13*	0.0286	0.4350	0.0658	2.38×10^{-110}
GaAHA06	*GaAHA13*	0.0298	0.4567	0.0652	6.06×10^{-115}
GrAHA02	*GrAHA05*	0.0222	0.6446	0.0345	5.80×10^{-147}
GrAHA06	*GrAHA14*	0.0977	0.6795	0.1439	4.18×10^{-116}
GrAHA07	*GrAHA09*	0.0289	0.3304	0.0875	8.25×10^{-79}
