# Supplementary material for: Identification and Analysis of the Plasma Membrane H+-ATPase Gene Family in Cotton and Its Roles in Response to Salt Stress
Source: Plants (Basel). 2024 Dec 16;13(24):3510. doi: 10.3390/plants13243510 (PMC11728463; doi:10.3390/plants13243510)
Supplement: Supplementary file 1 [file plants-13-03510-s001.zip › Table. S4.pdf]

Table S4. The primers used for qRT-PCR (5'-3') to verify the function of cotton PM H^{+}-ATPase genes in response to salt stress.

Primers	Nucleotide sequences (5'-3')
GhAHA09-F	CTTTTGTATTTGCTCTATTGCCATT
GhAHA09-R	CAGACAAAACAGTCGGCATAGC
GhAHA10-F	GTTCACTTCCTTCCATTCAATCC
GhAHA10-R	TCCACGCTCCGCAAACTTAT
GhAHA19-F	CATGACAATTTCCAAGGACAGAGT
GhAHA19-R	TCATGGCCCAGAAGAATAACAC
GhAHA21-F	TATTTTACTGAGAACATCCCGTGC
GhAHA21-R	CATGATAATGGGTTCCACATAAAAC
GhAHA33-F	ACGTCGAGTTCATGCTGTAATTG
GhAHA33-R	CGCCCTTCGTATTGTATCTGC
GhAHA34-F	CTGTTGCCTTTATTTGATCCTCC
GhAHA34-R	CCCAACAATGATGAAGAAGGATAC
GhAHA37-F	TCTTTGCTACTGGCATTGTCTTG
GhAHA37-R	GCTTCTTACGCCGAATTTATCC
GhAHA38-F	GAAGTTTGATTTCGCACCGTT
GhAHA38-R	TCCAAGCACAATGCCAGTAGAG
GhACTIN-F	ATCCTCCGTCTTGACCTTG
GhACTIN-R	TGTCCGTCAGGCAACTCAT
