# Supplementary figures and images for: Identification and Analysis of the Plasma Membrane H+-ATPase Gene Family in Cotton and Its Roles in Response to Salt Stress
Source: Plants (Basel). 2024 Dec 16;13(24):3510. doi: 10.3390/plants13243510 (PMC11728463; doi:10.3390/plants13243510)

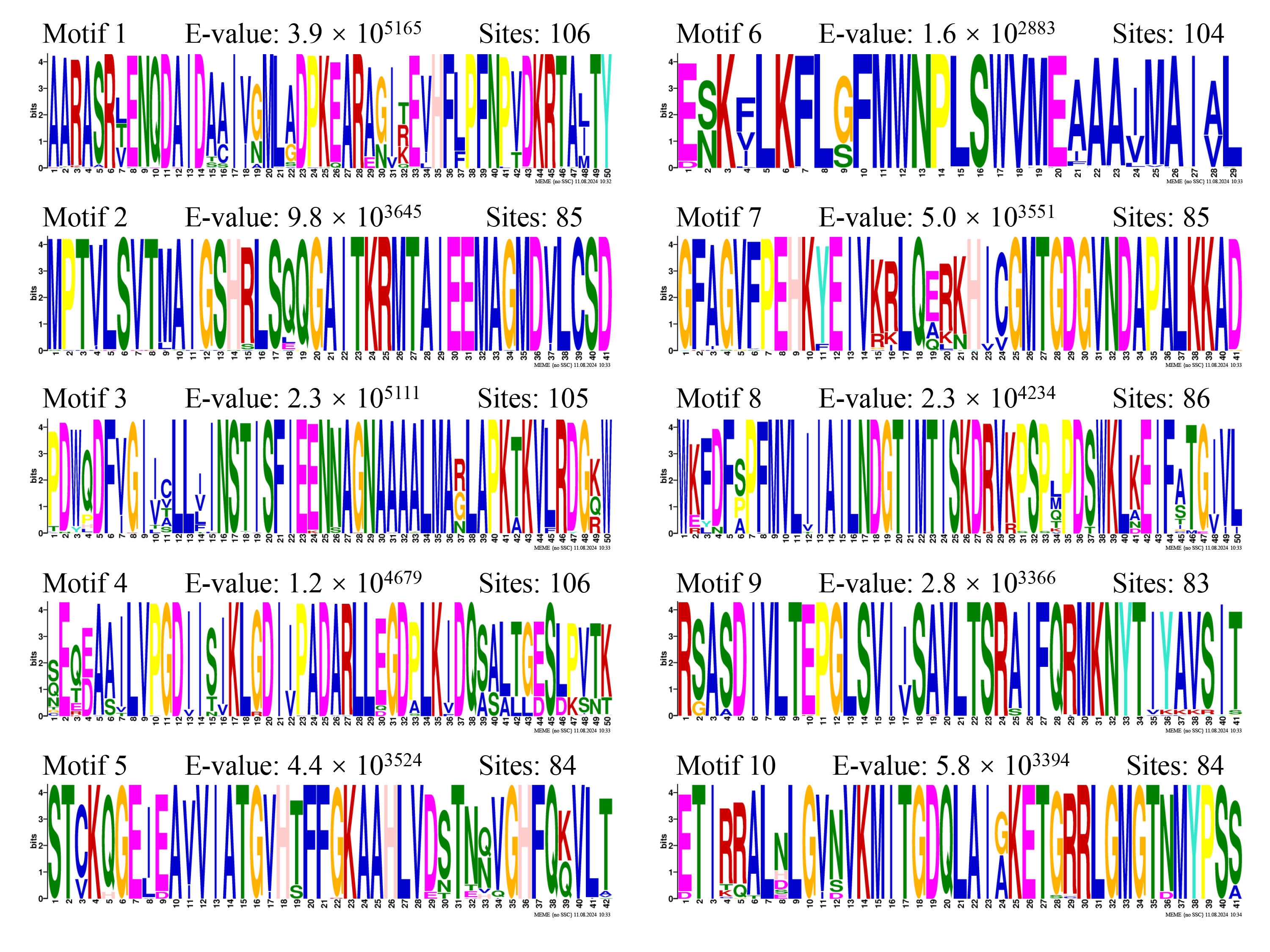

Supplement: Supplementary file 1 [file plants-13-03510-s001.zip › Fig.S1-revised.tif]

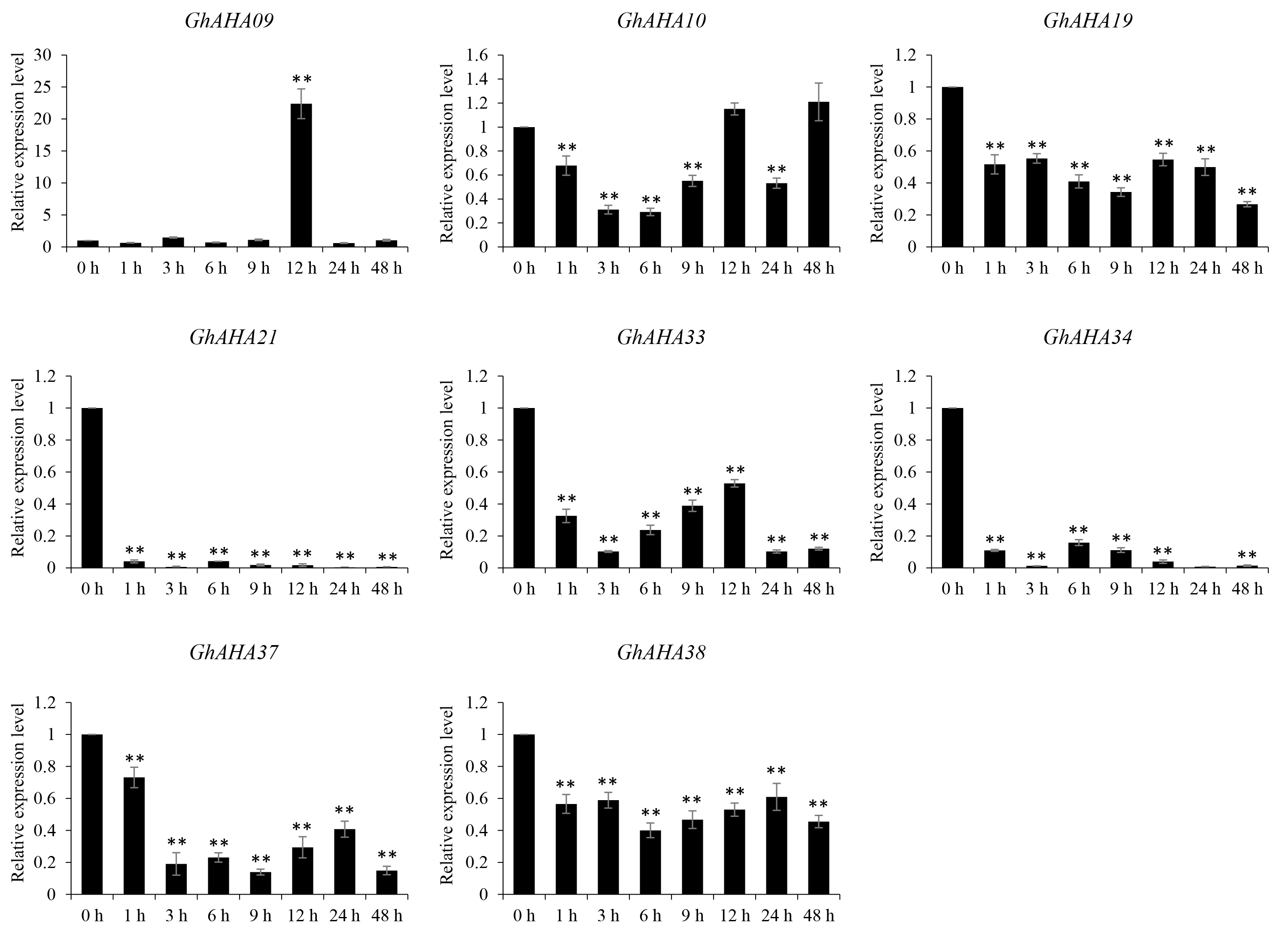

Supplement: Supplementary file 1 [file plants-13-03510-s001.zip › Fig.S2.tif]
